# Proposing a novel deep network for detecting COVID-19 based on chest images

**DOI:** 10.1038/s41598-022-06802-7

**Published:** 2022-02-24

**Authors:** Maryam Dialameh, Ali Hamzeh, Hossein Rahmani, Amir Reza Radmard, Safoura Dialameh

**Affiliations:** 1 Department of Computer Science, Shiraz University, Shiraz, Iran; 2 School of Computing and Communications, Lancaster University, Lancaster, UK; 3 Department of Radiology, Tehran University of Medical Sciences, Tehran, Iran; 4 School of Paramedical Sciences, Bushehr University of Medical Sciences, Bushehr, Iran

**Keywords:** Infectious diseases, Computational models, Data mining

## Abstract

The rapid outbreak of coronavirus threatens humans’ life all around the world. Due to the insufficient diagnostic infrastructures, developing an accurate, efficient, inexpensive, and quick diagnostic tool is of great importance. To date, researchers have proposed several detection models based on chest imaging analysis, primarily based on deep neural networks; however, none of which could achieve a reliable and highly sensitive performance yet. Therefore, the nature of this study is primary epidemiological research that aims to overcome the limitations mentioned above by proposing a large-scale publicly available dataset of chest computed tomography scan (CT-scan) images consisting of more than 13k samples. Secondly, we propose a more sensitive deep neural networks model for CT-scan images of the lungs, providing a pixel-wise attention layer on top of the high-level features extracted from the network. Moreover, the proposed model is extended through a transfer learning approach for being applicable in the case of chest X-Ray (CXR) images. The proposed model and its extension have been trained and evaluated through several experiments. The inclusion criteria were patients with suspected PE and positive real-time reverse-transcription polymerase chain reaction (RT-PCR) for SARS-CoV-2. The exclusion criteria were negative or inconclusive RT-PCR and other chest CT indications. Our model achieves an AUC score of 0.886, significantly better than its closest competitor, whose AUC is 0.843. Moreover, the obtained results on another commonly-used benchmark show an AUC of 0.899, outperforming related models. Additionally, the sensitivity of our model is 0.858, while that of its closest competitor is 0.81, explaining the efficiency of pixel-wise attention strategy in detecting coronavirus. Our promising results and the efficiency of the models imply that the proposed models can be considered reliable tools for assisting doctors in detecting coronavirus.

## Introduction

The early and accurate diagnosis of coronavirus disease of 2019 (COVID-19) plays a vital role in disease treatment and isolation. Current clinical methods such as nose swab PCR-test^1^ are insufficient in terms of both availability and accuracy, e.g., high rate of false-positive and low rate of sensitivity^2,3^. Apart from that, such methods are costly in terms of safety, human resources, and financial burdens^4^. Therefore, governments and hospitals cannot effectively tackle such a devastating pandemic, and novel screening methods should be adopted.

Chest radiography, such as chest X-ray (CXR) and CT computed tomography (CT), is another possibility to facilitate the process of screening COVID-19^5^; however, direct examination of such images by experts/specialists is tedious, time-consuming^3^, and possibly not accurate. In other words, it becomes tedious because, during the pandemic waves, hospitals are often faced with a massive number of patients and, hence, the amount of analysis/diagnosis that has to be done by experts/specialists will drastically increase^6^. Moreover, the early detection of COVID-19 by only visually monitoring chest imaging data would be almost inaccurate, as human visual systems cannot easily detect the patterns of lung infectious during the first days of the disease (supported by informal reported observations). Therefore, developing an automatic and precise image processing tool seems promising to address the problems mentioned above.

Due to the outstanding achievements of deep learning methods in a variety of domains^7–10^, researchers have proposed several interesting deep learning approaches for automatically classifying chest images as positive/negative COVID-19^11–14^. In brief, COVNET^15^ is one of the firstly proposed deep models for screening COVID-19 from the CT-images. COVNET consumes several lungs’ images at the same time and feeds each of which into a separated backend of RESNET-50^16^ whose weights are shared between its backends. The main limitation of COVNET, however, is a high level of computation with a low level of sensitivity. Hybrid-3D^17^ is another deep screening network that builds the 3D shape of lungs and then feeds both 2D and 3D inputs into two Densnet backbones^18^ and combines their predictions. However, its prediction is dependent on the accuracy of the estimated 3D shape of the lungs. Apart from that, the 3D estimation imposes a heavy computation, resulting in a lower screening speed. Unet++^19^, on the other hand, is computationally better than others. However, it does not satisfy the sensitivity requirements, i.e., the chance of false-negative is high. Table 3 summarizes the performances of these models on CT-COV19, which is a public benchmark. Detailed information about datasets and other related methods can be explored in^20–24^.

One of the main limitations of the current deep learning models is the size of datasets being used for learning the models, i.e., the number of publicly available training samples used for optimizing parameters. To the best of our knowledge, current models have been trained on small-sized datasets, mostly due to privacy concerns and the unavailability of COVID-19 CXR/CT images^25,26^. Consequently, it does lead to a lower generalization ability of the trained models^27^. Age and regional diversities are two other essential factors^28–30^, playing a vital role in the generalization of the learned models and preventing the models from the danger of overfitting. As elderly people are at a higher risk of being infected by the coronavirus, current public datasets of COVID-19 are often biased towards older people, resulting in a lower chance of generalization for younger patients. Additionally, people of different regional backgrounds are not necessarily common in their lung functioning, respiration abilities, and several other breathing factors^31^. Hence, if a dataset is built based on the samples provided by one hospital/city, the models learned on such datasets would be very likely to become biased toward that specific region. The lack of sensitivity in predictions is another critical limitation of the current deep neural models, causing less reliable detection. In other words, it is currently very likely to predict a positive sample as a negative, particularly during the incubation period of disease when there are no clear patterns of infection in the lungs.

This study aims to overcome the limitations above, resulting in more accurate and reliable deep neural models for being a helpful side-tool in screening COVID-19. Accordingly, this study, firstly, builds a publicly available CT-scan dataset of COVID-19, consisting of 13k CT-images captured from more than 1000 individuals. The images are collected from four regions with entirely different climate conditions. A wide range of age diversities has also been included, ages varying from 19 to 73. Additionally, images are saved at a high level of quality. Overall, the proposed dataset, named CT-COV19, provides a reliable set of CT-scan images for the researchers to develop more accurate and general models. Secondly, the present study suggests a novel deep neural model, i.e., Deep-CT-Net, trained on the proposed CT-COV19 dataset and provides baseline results. Deep-CT-Net benefits from a simple but accurate architecture, enabling the possibility of early screening the infection patterns of COVID-19 from the CT-images of lungs. More precisely, the proposed model takes advantage of pyramidal attention layers^32^, providing pixel-wise attention on top of the extracted high-level features and, consequently, enabling the whole model to accurately detect COVID-19 even when there are less symptoms of the disease in the lungs. The pixel-wise attention empowers the final model to detect more positive cases whose primary swabs are negative. Furthermore, having no heavy pre-processing steps, such as lungs segmentation^17,33^, is another virtue of the proposed network, enabling the model to detect COVID-19 in a lower computational time. This property is particularly desirable during the waves of COVID-19, as the detection process becomes much faster than those models containing the pre-processing steps.

Extensive experiments on several benchmarks of COVID-19 are conducted, and the results are compared with several state-of-the-art methods^15,17,19,34–37^. Moreover, a transfer-learning version of Deep-CT-Net, i.e., Deep-CXR-Net, is further developed to detect COVID-19 based on CXR images. The choice of transfer learning enables the network to learn from unlabeled CXR images and, therefore, adjusts the weights by a small set of labelled CXR images. Additionally, the results of Deep-CXR-Net are compared with several related methods, e.g., Refs^11,13,38^.

## Results

This section provides a detailed explanation of CT-COV19 and reports the performance results of proposed models over several popular benchmarks, including CT-COV19. The performance criteria are described in Ref.^39^. Additionally, Li’s procedure^40^ is applied as a post-secondary statistical test over the results in terms AUCs.

### CT-COV19: a public CT-scan dataset for COVID-19

Approving by the institutional review board (IRB), this section describes the details of our publicly available CT-scan dataset named CT-COV19 for screening COVID-19. CT-COV19 consists of 13k CT-images of the lungs and is obtained by a non-contrast chest CT, in which the reconstructions of the volume are set at 0.3 to 1 mm slice thickness. The images are taken from more than 1000 randomly selected male and female individuals, i.e., $Male: 59\%, Female: 41\%$. Among the patients, 500 cases were infected with COVID-19. An RT-PCR test was performed to confirm their infections with COVID-19. Moreover, the age of individuals ranges from 19 to 73. Therefore, CT-COV19 is diverse in terms of both gender and age groups. The regional diversity has also been included in CT-COV19, as it has been collected from four different regions with diverse climates. It is worthwhile noting that the collected data are anonymous, and privacy concerns are satisfied.

One of the main advantages of CT-COV19 is the number of COVID-19 samples, which is the most considerable size among the publicly available datasets of COVID-19 by far. Another aspect of CT-COV19 is the existence of samples from other pneumonia, providing learning algorithms with an opportunity to distinguish between infections caused by COVID-19 and other lung diseases. Table 1 provides a brief comparison between CT-COV19 and several other similar datasets, explaining the quantitative superiority of CT-COV19 to others.

**Table 1:** A brief comparison between several publicly available datasets for CT images of COVID-19.

Dataset	# COVID-images	# COVID-patients	Availability
Jun et al^41^	20	N.A.	Y
Covid-19-SEG^42^	100	60	Y
Lung-ct-scan^43^	15 k	95	Y
COVID-CT^36^	349	216	Y
SIRM COVID-19 Database^44^	100	60	Y
CT-COV19 (this study)	8500	500	Y

**Table 2:** CT-COV19 is divided into three parts: Train, Test, and Validation.

Data-part	COVID-19	Other pneumonia	Normal	Total
Train	6120	543	3060	9723
Validation	680	60	340	1080
Test	1700	151	851	2702
Total	8500	754	4251	13,505

Each row illustrates how many samples from each class, i.e., COVID-19, other pneumonia, and normal, are included in each part. Each column shows the distribution of classes over the parts.

**Figure 1:**
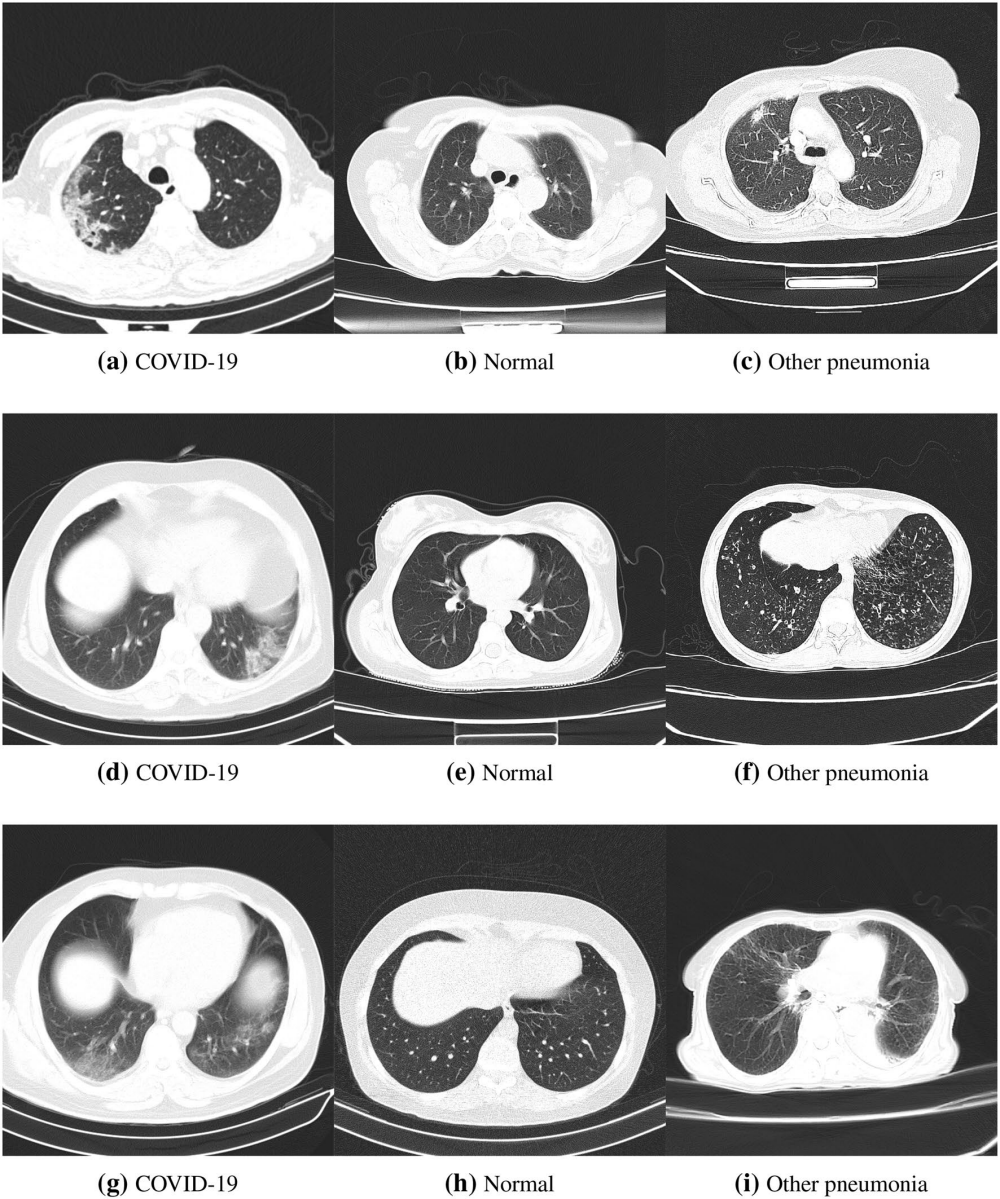
Several different instances of the proposed dataset, i.e., CT-COV19: each column provides several examples of each class presented in the dataset.

CT-COV19 consists of CT-scan images from three different labels, including COVID-19, other pneumonia, and normal with the ratios of $61.5\%, 5.8\%, 32.7\%$, respectively. Although the number of samples belonging to the class of other pneumonia is small, one can easily find plenty of such samples via the Internet. In this study, we deliberately merge the labels of other pneumonia with all samples of normal class and consider the merged set as the normal class. Therefore, CT-COV19 is generally a two classes dataset. This dataset, which now has two classes, i.e., COVID and Normal, is further randomly divided into train, validation, and test parts with ratios of 70%, 10%, and 20%, respectively. Table 2 summarizes this division. The minimum and maximum heights/widths of images are respectively $484\times 484$ and $1024\times 1024$. Additionally, the minimum resolution of the images is 150dpi, and the bit depth is 24. Figure 1 provides several samples of this dataset.

### The results of Deep-CT-Net

**Figure 2:**
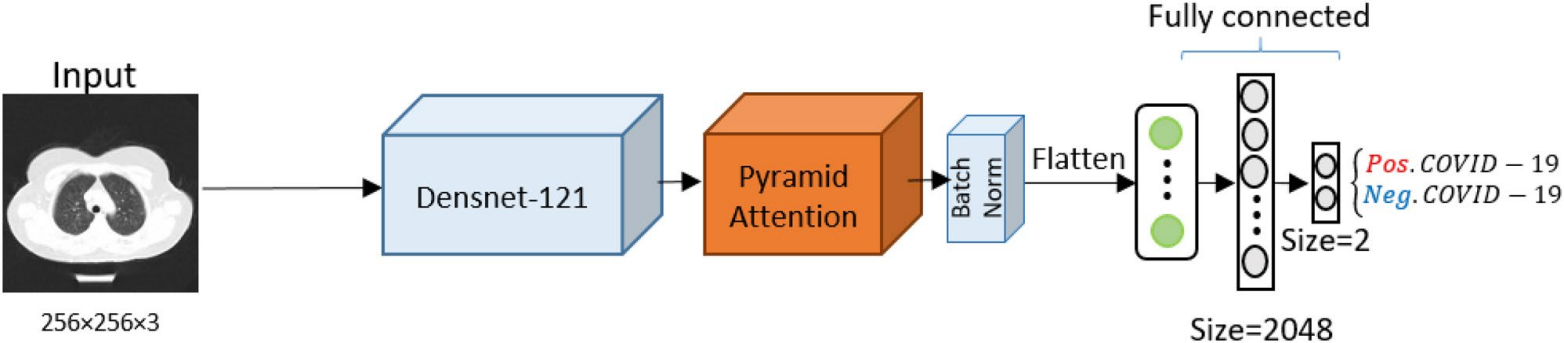
(Deep-CT-Net) The figure depicts a graphical workflow of the proposed Deep-CT-Net for detecting COVID-19 based on the CT-image data. More information about the components used in the network is provided in Supplementary Figure 1.

This subsection reports the empirical results of the proposed deep neural architecture (Deep-CT-Net), which can classify CT-scan images into two classes: positive-COVID and negative-COVID. Figure 2 depicts the workflow of Deep-CT-Net.

Deep-CT-Net is assessed through several experiments. In the first experiment, we evaluate the performance of Deep-CT-Net on two datasets, i.e., CT-COV19 (proposed in this study) and COVID-CT^36^, and compare its performance against several related deep network models^15,17,19,35^. In brief, COVID-CT has a small number of samples with a lower image quality compared to CT-COV19. Figure 6 depicts several images of this dataset.

As shown in Tables 3 and 5, and Fig. 4, Deep-CT-Net achieves an area under curves (AUC) of 0.886 and 0.899 on each dataset. Also, the rates of precision, sensitivity, and F-measure for Deep-CT-Net are respectively 0.720, 0.858, 0.783 on CT-COV19 and 0.884, 0.905, 0.894 on COVID-CT. The performances of other models are summarized as follows:

With CT-COV19, the obtained precision, sensitivity, F-measure, and AUC are respectively equal to: 0.750, 0.810, 0.779, 0.842 in case of COVNET, and 0.769, 0.75, 0.759, 0.804 in case of DL-system, and 0.808, 0.797, 0.802, 0.843 in case of Hybrid-3D, and 0.724, 0.746, 0.735, 0.826 in case of Unet++. With COVID-CT, the obtained precision, sensitivity, F-measure, and AUC are, respectively, equal to: 0.897, 0.886, 0.892, 0.886 in case of xDNN, and 0.97, 0.762, 0.853, 0.824 in case of its Baseline, and 0.817, 0.85, 0.833, N.A., in case of Modified SqueezeNet, where N.A. stands for being not available. Moreover, the p-values obtained by Li’s procedure are: 0.05 for Hybrid-3D, and 0.0376 for the rest. Li’s procedure rejects those hypothesis whose p-values are lower or equal to 0.0376.

The next experiment assesses the generalization ability of Deep-CT-Net. Accordingly, Deep-CT-Net is trained on CT-COV19 but tested on COVID-CT dataset^36^ without applying any fine-tuning or post-processing step. Table 4 reports the obtained results of Deep-CT-Net on COVID-CT dataset and provides a comparison with the baseline methods reported in Ref.^36^. As the table reports, Deep-CT-Net achieves $AUC=0.92$ and F-measue = 0.801 (Table 5).

The final experiment is conducted to have a better view of the functionality of Deep-CT-Net. Figure 5 depicts Class Activation Mapping (CAM)^45^ for a test sample taken from COVID-19, visualizing the attention regions inside the lungs. As shown in this figure, the attention regions detected by our proposed Deep-CT-Net are precisely related to COVID-19 symptoms, i.e., the highlighted lungs’ regions in red.

**Figure 3:**
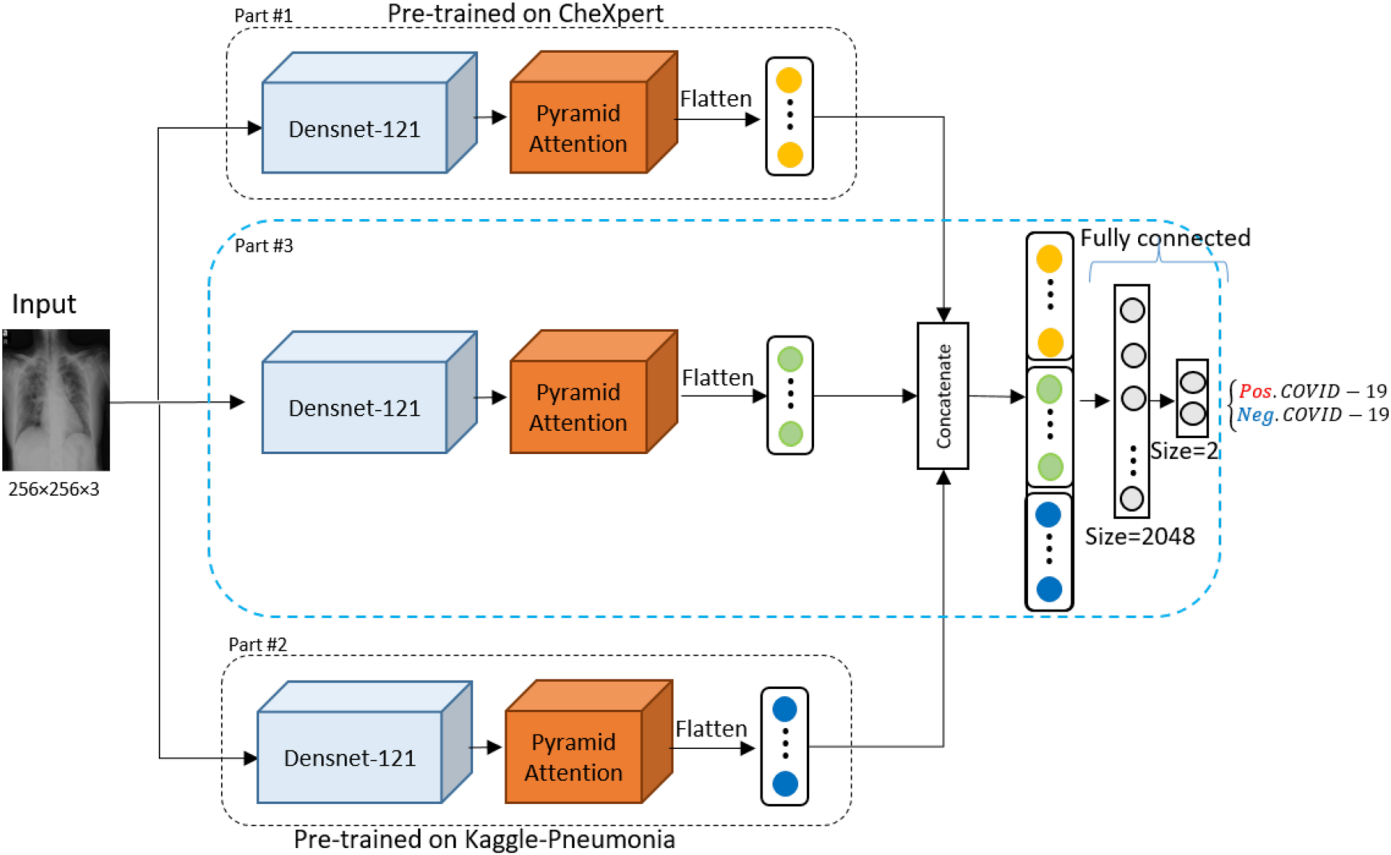
(Deep-CXR-Net) A graphical workflow of Deep-CXR-Net for screening COVID-19 based on the CXR data is depicted in the figure. Overall, Deep-CXR-Net consists of two pre-trained parts, extracting extra features to be concatenated with the features generated by the second part. The concatenated features are then passed through the fully connected layers to predict the label. More information about the components used in the network is provided in Supplementary Figure 1.

**Table 3:** Comparing the performance of each model over the proposed CT-COV19 dataset.

Method	Precision	Sensitivity	F-measure	AUC
COVNET^15^	0.750	0.810	0.779	0.842
DL-system^35^	0.769	0.750	0.759	0.804
Hybrid-3D^17^	0.808	0.797	0.802	0.843
Unet++^19^	0.724	0.746	0.735	0.826
Deep-CT-Net (this study)	0.720	0.858	0.783	0.886

Each method is trained and tested over the CT-COV19, and the results are reported as well.

**Figure 4:**
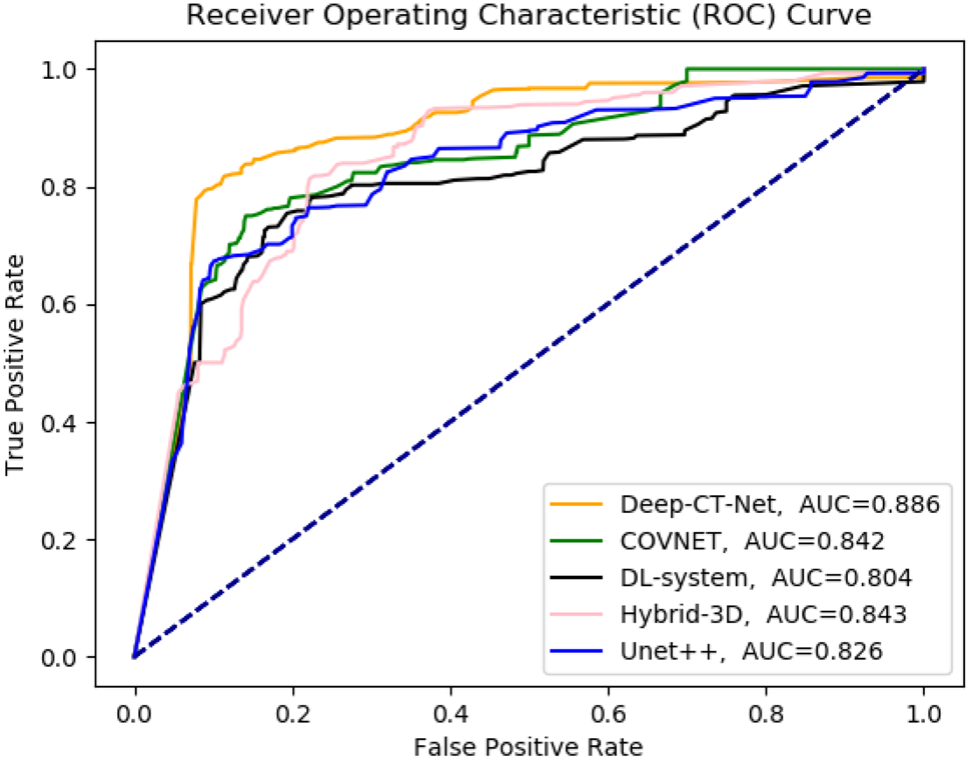
The obtained receiver operating characteristic (ROC) curves over the proposed CT-COV19 dataset. The figure plots the ROC curve obtained by Deep-CT-Net and several related models.

**Table 4:** An Evaluation of the generalization ability of Deep-CT-Net: the proposed Deep-CT-Net was trained on our proposed CT-COV19 dataset and tested on COVID-CT without any fine-tuning.

Method	F-measure	AUC	Accuracy
DenseNet-169	0.760	0.901	0.795
ResNet-50	0.746	0.864	0.774
Deep-CT-Net (trained on CT-COV19)	0.801	0.92	0.86

Other models in the table have been trained and tested on CT-COV19. The results show the generalization ability of the proposed method.

**Table 5:** Comparing the performance of Deep-CT-Net against other related models on COVID-CT.

Method	Precision	Sensitivity	F-measure	AUC
xDNN^37^	0.897	0.886	0.892	0.886
Baseline^36^	0.970	0.762	0.853	0.824
Modified SqueezeNet^34^	0.817	0.850	0.833	N.A.
Deep-CT-Net	0.884	0.905	0.894	0.899

All models in this table are trained and tested on COVID-CT.

**Figure 5:**
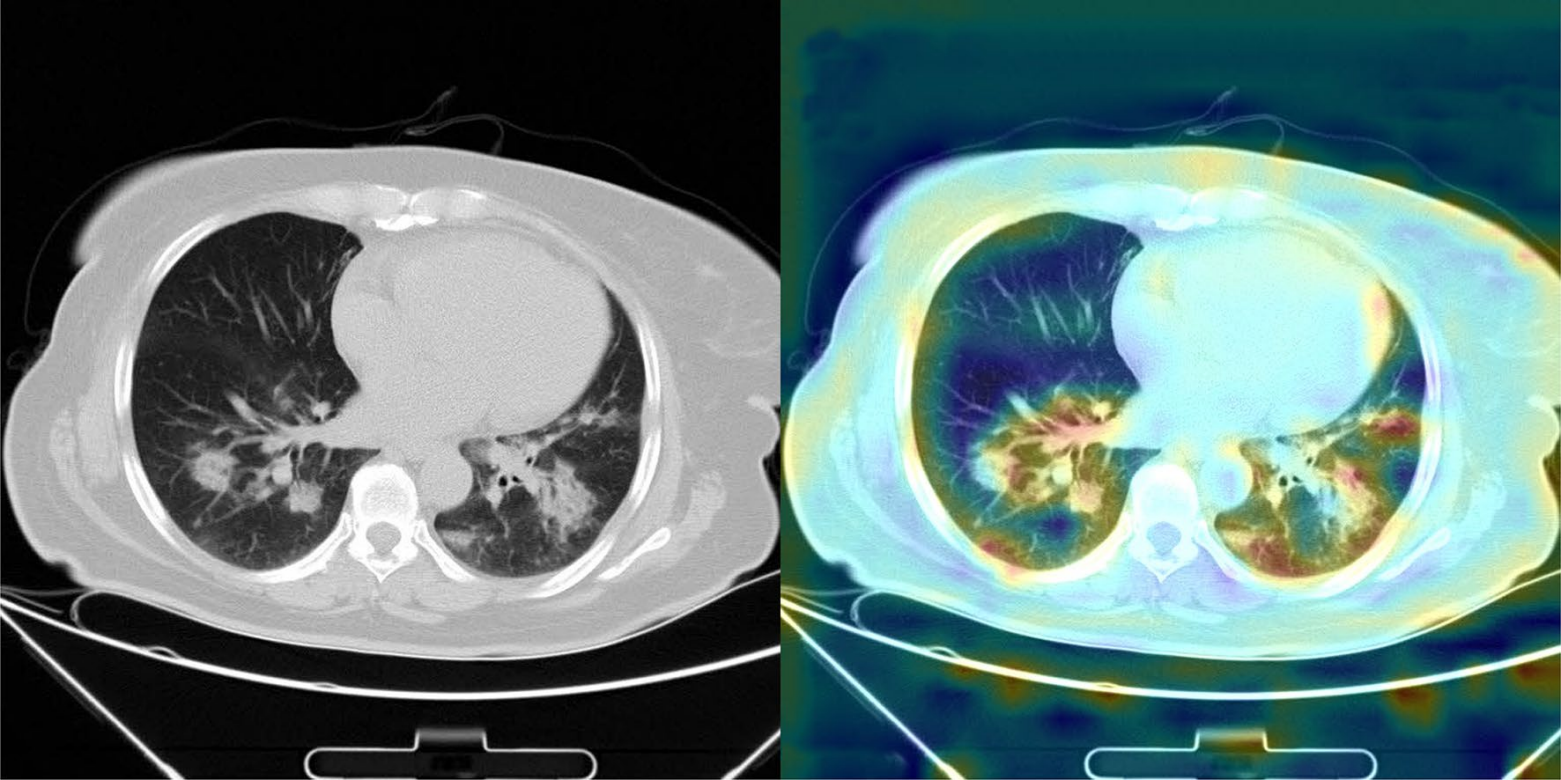
Plotting the results of Class Activation Mapping (CAM) for a CT-image instance of COVID-19 (left) based on Deep-CT-Net: The CAM image (right) highlights the class-specific discriminative/attention regions. The highlighted areas inside of the lungs are the discriminative regions for screening COVID-19.

**Figure 6:**
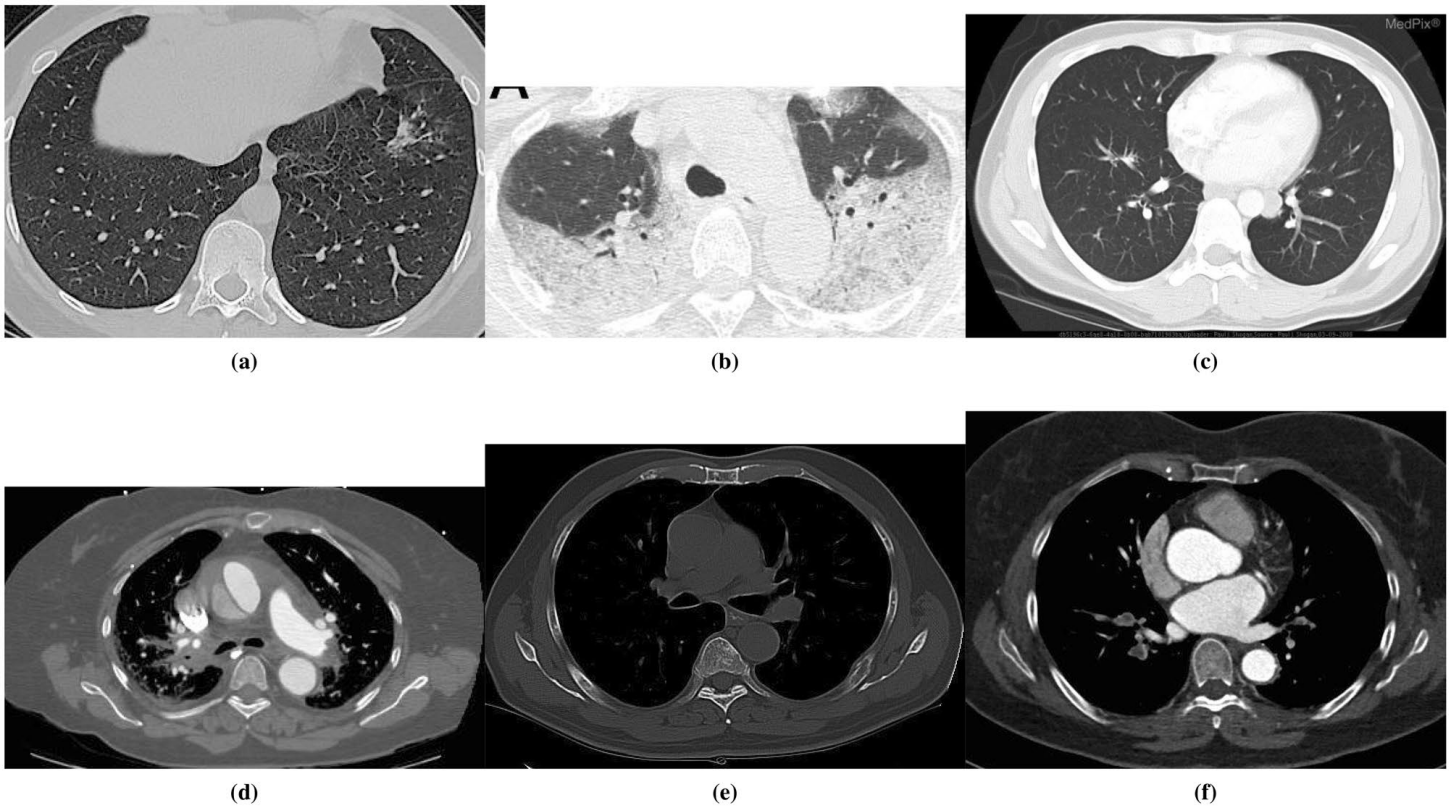
Several instances of COVID-CT dataset: The images come with diverse illumination, format, and shapes.

### The results of Deep-CXR-Net

**Figure 7:**
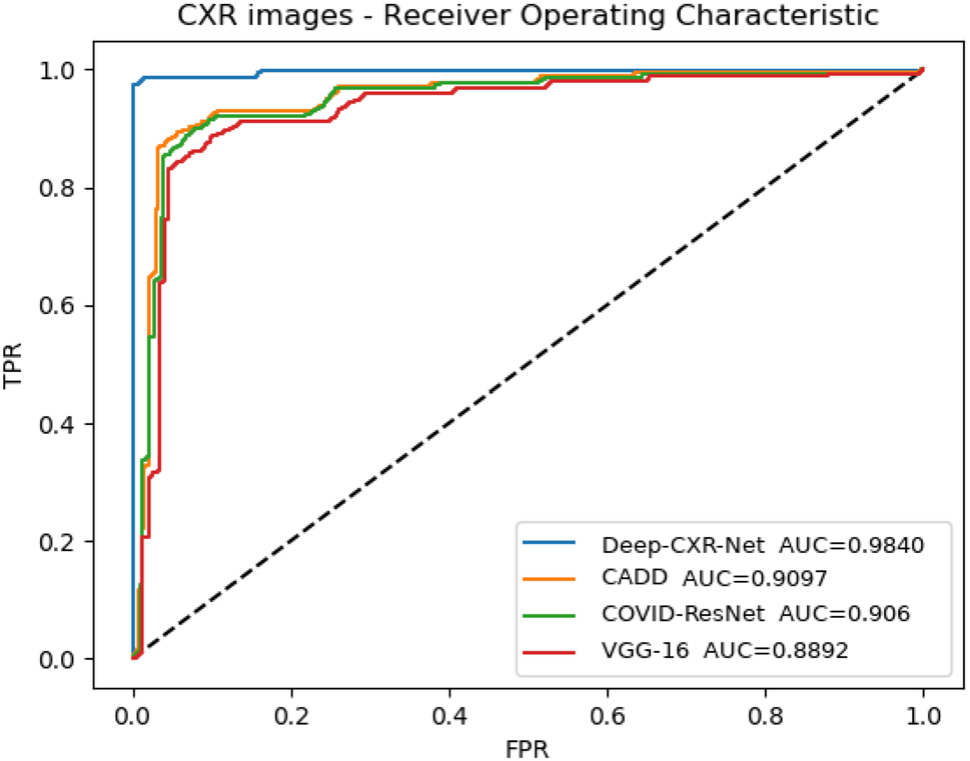
The obtained ROC curves for ieee8023 dataset. The figure plots the ROC curve obtained by Deep-CXR-Net and compares it with that of several related models.

**Figure 8:**
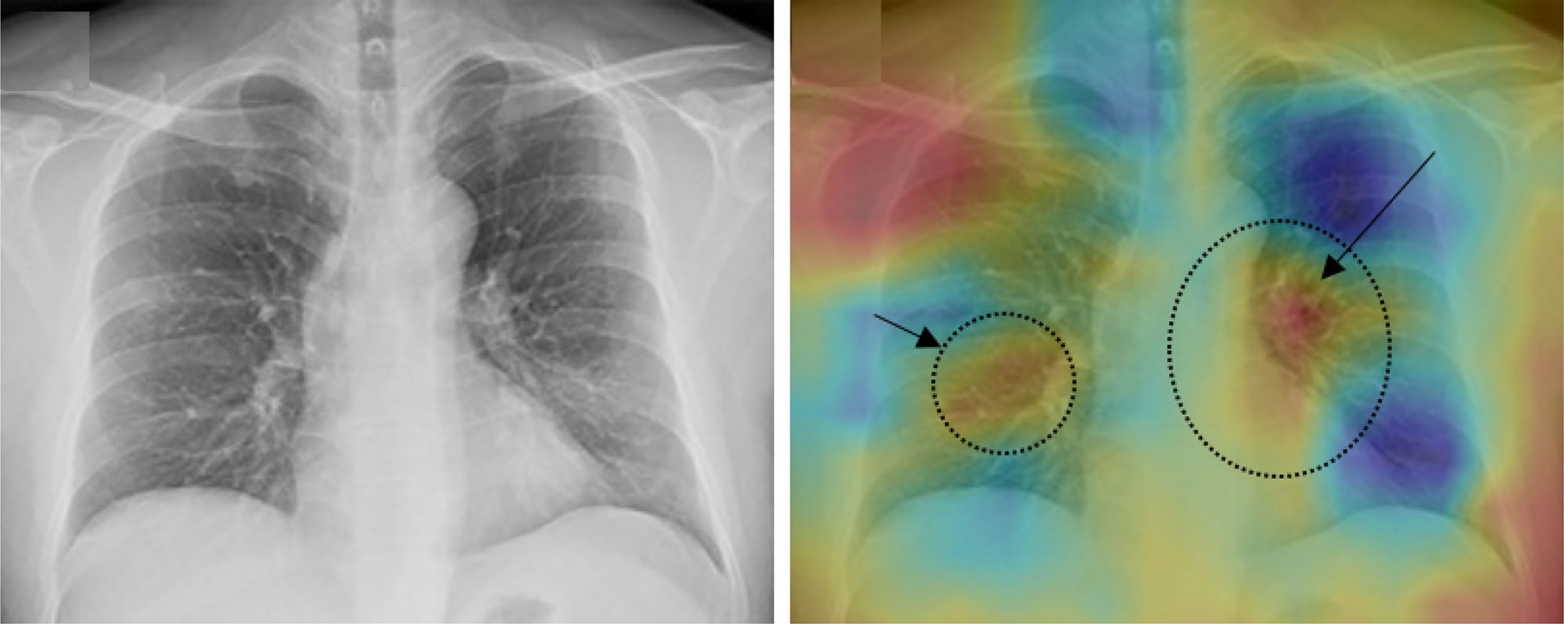
(Deep-CXR-Net) Plotting the Class Activation Mapping (CAM) results for Deep-CXR-Net: The CAM highlights the class-specific attention regions. All images belong to positive COVID-19 cases. The dotted regions inside of the chests are discriminative areas.

The obtained results of Deep-CXR-Net, which is the CXR extension of Deep-CT-Net, and its workflow is depicted in Fig. 3, are reported in this subsection. To have a reliable comparison, we randomly divided ieee8023^46^ to train and test sets with the proportions of 70% and 30%, respectively. Then, we added 100 more CXR images of other pneumonia to the test set. This set of additional CXR data helps to evaluate the performance of each method in terms of false-positive rates. Table 6 compares the obtained screening results of Deep-CXR-Net to other related methods^11,13,38^. As shown in Table 6 and depicted in Fig. 7, Deep-CXR-Net achieves an area under the curve (AUC) of 0.9839. Additionally, the corresponding precision, sensitivity, and F-measure rates are equal to 0.9872, 0.9824, 0.9848. The obtained precision, sensitivity, F-measure, and AUC for the other related models are, respectively, equal to: 0.9431, 0.915, 0.924, 0.9207 in case of CAAD, and 0.926, 0.916, 0.92, 0.9162 in case of COVID-ResNet, and 0.898, 0.894, 0.896, 0.8892 in case of VGG16. Moreover, Fig. 7 compares the obtained receiver operating characteristic (ROC) curves for each method. It is important to note that the same experimental settings have been used for other methods. Additionally, Fig. 8 depicts the results of class activation map for a CXR-sample of COVID-19. The dotted areas are those regions of the lung that play an essential role in classifying this sample as COVID-19.

**Table 6:** The obtained results over the CXR dataset, i.e., ieee8023.

Method	Precision	Sensitivity	F-measure	AUC
CAAD^11^	0.9431	0.9150	0.9240	0.9207
COVID-ResNet^13^	0.9260	0.9160	0.920	0.9162
VGG16^38^	0.8980	0.8940	0.8960	0.8892
Deep-CXR-Net (this study)	0.9872	0.9824	0.9848	0.9839

## Discussion

The experimental results certify the superiority of the proposed models in comparison with similar studies. Overall, both Deep-CT-Net and Deep-CXR-Net achieve higher sensitivity rates than the others, i.e., 0.905 for Deep-CT-Net and 0.9824 for Deep-CXR-Net. This observation certifies that the pyramid attention layer embedded in Deep-CT-Net can successfully detect a diverse range of infection patterns caused by COVID-19. Additionally, although the results show lower precision for Deep-CT-Net, its overall score (F-measure) is better, enabling the proposed models to be used in clinical diagnostics. Finally, the main implications of this study can be summarized as follows:

- the proposed deep learning models have practical implications, and they can be used as an assistant in diagnosing coronavirus.
- applying the pyramidal attention layers plays a significant role in detecting coronavirus accurately. Future deep neural models can take advantage of this layer, increasing the overall performance of models.
- Deep-CXR-Net offers a transfer learning approach for training deep neural models while less data is available for training.

To discuss more in related methods and compare their main ideas, COVNET^15^ combines several ResNets^16^ with shared weights along with a series of CT images, in which each ResNet consumes one CT image. Finally, a pooling layer aggregates their outputs. Although the weights are shared, the computational time/cost is high in practice. COVNET reports a sensitivity of 0.81 and an AUC of 0.842 over CT-COV19. Similarly, DL-system^35^ is computationally complex. It uses three main stages: lungs’ segmentation, segment suppression, and prediction, consuming high level of computation. Moreover, it uses 3D convolutions, which even need more computational power. Another limitation is that the prediction accuracy becomes dependent on the segmentation accuracy, causing this model to be not operational in practice. Overall, DL-system reports the lowest accuracy in prediction, i.e., 0.75 of sensitivity and 0.804 of AUC over CT-COV19. Hybrid-3D^17^ works in a similar approach to DL-system. It first segments lungs and then applies Densnet-121 for classification. Although its performance is better than DL-system, it still suffers from the same limitations of DL-system. Hybrid-3D reports a sensitivity of 0.797 and an AUC of 0.843 over CT-COV19. CAAD^11^, which is a CXR model, suggests an anomaly detection loss on an 18-layer residual convolutional neural network, enabling the model to increase the probability of positive class in prediction. Other CT/CXR models such as Unet++, xDNN, and VGG16 are popular deep architectures in other tasks, and they have no special contribution for detecting COVID-19. Overall, Unet++ reports a sensitivity of 0.746 and an AUC of 0.826 over CT-COV19.

This work is significant from three main perspectives. First, we built a publicly available dataset of CT-images of COVID-19 that is large and diverse enough to train reliable models and, therefore, can be considered for training and evaluation in future studies. Second, the proposed deep neural networks can extract pixel-wise information accurately and, thus, detect COVID-19 with higher accuracy. That is why Deep-CT-Net and Deep-CXR-Net achieve higher rates of sensitivities than other related methods. For instance, the closest method to Deep-CT-Net, in terms of sensitivities on CT-COV19, is COVNET with a value of 0.81, while that of Deep-CT-Net is 0.858. The same observation can be seen from the obtained results over COVID-CT, in which the sensitivity rate achieved by Deep-CT-Net is 0.905, while that of the closest related method, i.e., xDNN^37^, is 0.886. Third, the proposed Deep-CXR-Net, which is the CXR extension of Deep-CT-Net, is able to be trained on small-sized CXR datasets and efficiently compensate for the lack of enough CXR data of COVID-19. Compared with other CXR-based deep models, we found that the choice of using additional features results in much better performances for unseen CXR data and significantly reduces the rate of false-positive. In contrast, other methods have shown higher false-positive rates in their predictions, i.e., they wrongly tend to predict samples of other pneumonia as COVID-19.

The results of class activation map (CAM) for each model are also depicted in Figs. 5 and 8, which are derived from Deep-CT-Net and Deep-CXR-Net respectively. The highlighted regions (red colour inside the lungs) in the CAM results depict those parts of the lungs where the COVID pneumonia appears. Interestingly, we can see from the figures how well the models could detect the infectious regions of COVID.

One of the main advantages of the proposed models is their efficiency in terms of computational complexities, allowing them to be used in ordinary computing systems of hospitals. More accurately, the size of models is not huge, particularly for the Deep-CT-Net, and also, there are no complex pre-processing steps. For instance, the models in Refs.^17,33^ are based on complex lung segmentation steps, imposing more computational cost in practice and limiting the final screening results to the segmentation accuracy. Being efficient and accurate, the proposed models have the potential to be used in hospitals’ emergency rooms. Accordingly, a regular computer equipped with an ordinary Nvidia graphic card can be connected to the imaging system, either CT-scan or CXR, making the prediction in a fraction of a second. In contrast, deep neural models with complex network or overhead processing need advanced graphic cards and computers to predict online, often not applicable in all hospitals due to the lack of computing hardware.

On the other hand, this study has faced several limitations. First, more experiments should be conducted to examine the generalization ability of Deep-CT-Net on the datasets of different medical centers; second, although the attention layers increase the sensitivity rate, the precision rate decreases. Finally, the proposed models only detect positive and normal classes and are not able to quantify other pneumonia, e.g., bacterial pneumonia. Future studies may address such limitations.

In conclusion, this study proposed a publicly accessible benchmark of COVID-19 CT images of lungs, allowing further studies to build more general models. We further proposed a baseline model called Deep-CT-Net, benefiting from a pyramidal attention layer that helps to extract discriminative pixel-wise features. Moreover, we extended our model for the case of CXR images of lungs using a transform learning strategy, enabling it to be trained on a small number of samples. The experimental results show that: (1) the choice of pyramidal attention layers can significantly increase the sensitivity rate, increasing the overall prediction metrics, i.e., AUCs, and (2) the proposed Deep-CT-Net is likely to have more false positives in favour of having a lower rate of false negatives. Overall, we found that the pixel-wise features extracted by pyramidal attention layers can significantly enhance the prediction performance of deep neural models.

As for future work, we plan to design a deep neural decoder for extending the current models to accurately segment the infected parts of the lungs with COVID-19, resulting in discovering biomarkers for COVID-19. This result could further be used to categorize the infection patterns of COVID-19, providing valuable data sources for revealing the unknown aspects of the virus and eventually being helpful in medical prescriptions. Additionally, Deep-CT-Net provides a baseline result over the proposed CT-COV19 dataset, and there is still room for developing more accurate deep models.

## Methods

### Data pre-processing

The data pre-processing stage consists of five consecutive steps. After resizing all the images into a similar resolution, e.g., $512\times 512$, the first step is applying random data augmentation techniques, including a random rotation in a degree of [−15,15], and a random translation in a range of [−0.05,0.05]. The second step is performing a histogram equalization, which adjusts the contrast of a CT-scan image by modifying the intensity distribution of the histogram. The third step is fixing the aspect ratio of images (or image resizing) to a size of $256\times 256\times 3$. Afterward, a Gaussian blur filtering with a window-size of 3 is used for image smoothing. Finally, images are normalized by subtracting from their mean and divided by their standard deviation.

### Network architecture

This subsection introduces the proposed deep neural architectures, i.e., Deep-CT-Net and Deep-CXR-Net, which can accurately classify CT-scan images into two classes: positive-COVID and negative-COVID. The following paragraphs explain their architectures and other implementation details to make them reproducible.

Figure 2 depicts the workflow of Deep-CT-Net. As it is shown, Deep-CT-Net consists of three main parts. The first part applies Densnet-121^18^ as the backbone, extracting high-level features from the input raw CT-images. The second part performs a pyramid attention layer^32^ over the extracted high-level features to maximize pixel-wise feature extraction, allowing the model to detect COVID-19 even during the first days of infection. A batch normalization layer, which standardizes the input to the next layer, is then used to avoid internal covariance shifts^47^ and have a smoother objective function^48^. Finally, the last part flattens the output of the previous part to be fed into fully connected layers for prediction. The backward pass then updates the weight parameters of all three components using the Adam optimizer^49^ with a learning rate of 1e-5 to minimize a binary-cross-entropy loss.

The architecture of Deep-CXR-Net, which is a transfer learning extension of Deep-CT-Net for screening COVID-19 based on CXR-images of lungs, is depicted in Fig. 3. The choice of transfer learning allows the Deep-CXR-Net to learn from a small set of labelled CXR COVID-19 images while providing a high level of generalization ability. More accurately, the proposed Deep-CXR-Net consists of three main parts, where the first two parts are independent pre-trained models respectively on two large-sized datasets of non-COVID diseases of lungs, i.e., CheXpert^50^ and Kaggle-Pneumonia^51^. We consider these parts as two black-box functions whose inputs are CXR images, and the outputs are vectors of high-level features. More precisely, for a given CXR image, the output of the first part is a six-dimensional vector whose entries are the likelihood of six certain lung diseases, documented in Ref.^50^. Besides, the output of the second part is a two-dimensional vector, representing the likelihood of having pneumonia or not. These additional features compensate for the scarcity of CXR data in COVID-19, increasing the generalization ability of the Deep-CXR-Net. The last part, i.e., Part 3, is another deep network, concatenating all the extracted features, i.e., the extracted features of itself and those from parts one and two. This concatenation is the point where the idea of transfer learning comes to play. More precisely, the additional concatenated features provided by Parts 1 and 2 compensate for the lack of having enough CXR training samples of COVID-19, resulting in a high level of generalization during the test time. We use ieee8023^46^ as a COVID-labeled dataset to train the parameters of the third part.

Additionally, a certain number of image augmentation techniques, such as rotation and translation (explained above), have also been applied in the learning phase. As parts 1 and 2 are pre-trained models, the backward pass only updates the weight parameters of the third part using the Adam optimizer with a learning rate of 1e-5 to minimize the binary-cross-entropy loss. As Fig. 3 shows, the backbone applied in all three parts is DensNet-121. Similar to Deep-CT-Net, the proposed Deep-CXR-Net uses a pyramidal attention layer^32^ to provide pixel-wise attention on high-level features, enabling the whole model to effectively detect COVID-19 cases even when there are small clues of the disease in lungs.

### Statement

All the experiments, as well as methods, were carried out under relevant guidelines and regulations. All protocols used in the experiments were approved by Shiraz University. The process of collecting the CT-data, i.e., CT-COV19 was approved by the ethics committee of the Shiraz University of Medical Sciences. Informed consent was obtained from all subjects.

## Supplementary Information

Supplementary Figure 1.

## Data Availability

Our proposed dataset, i.e., CT-COV19, is publicly reachable via this link: https://github.com/m2dgithub/CT-COV19.git.
