# Supplementary material for: Proposing a novel deep network for detecting COVID-19 based on chest images
Source: Sci Rep. 2022 Feb 24;12:3116. doi: 10.1038/s41598-022-06802-7 (PMC8873454; doi:10.1038/s41598-022-06802-7)
Supplement: Supplementary file 1 — Supplementary Figure 1. [file 41598_2022_6802_MOESM1_ESM.pdf]

Proposing a Novel Deep Network for Detecting COVID-19 Based on Chest Images

Maryam Dialameh[1,*], Ali Hamzeh[1], Hossein Rahmani[2], Amir Reza Radmard[3], and Safoura Dialameh[4]

[1]Department of Computer Science, Shiraz University, Shiraz, IRAN
[2]School of Computing and Communications, Lancaster University, UK
[3]Department of Radiology, Tehran University of Medical Sciences, Tehran, Iran
[4]School of Paramedical Sciences, Bushehr University of Medical Sciences, Bushehr, Iran
[*]corresponding.4tiamo4@gmail.com

1 APPENDIX

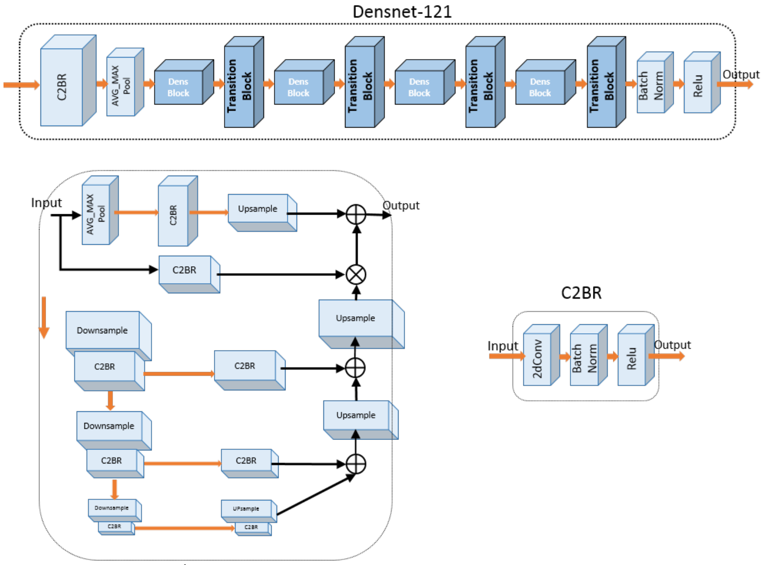

Supplementary Figure 1. A graphical explanation of the components being used in the proposed methods.
